# Subclinical Variation in Mental Health Shapes the Dynamics of Everyday Behaviour

**DOI:** 10.1177/17470218261424186

**Published:** 2026-02-05

**Authors:** Amber J. Brown, Margaret C. Macpherson, Lynden K. Miles

**Affiliations:** 1 School of Psychological Science, University of Western Australia, Perth, WA, Australia; 2 School of Psychological Sciences, Macquarie University, NSW, Australia

**Keywords:** dynamical systems theory, everyday behaviour, social anxiety, autism

## Abstract

Everyday behaviour is comprised of myriad components that must be seamlessly coordinated for action to be effective. Individual differences, specifically variation in mental health symptoms, influence how this challenge is navigated, however, their impact in naturalistic settings remains unclear. Adopting a dynamical systems perspective, here we examined whether subclinical variation in symptoms associated with social anxiety disorder (SAD) and autism spectrum disorder (ASD) modulate individual movement dynamics during an everyday activity – walking on a university campus. Participants (*n* = 93) completed two walking trials, the second of which included an additional distractor task that they were either told to ignore or attend to. Gait dynamics were captured unobtrusively and assessed at both local (i.e., moment-to-moment) and global (i.e., time invariant) levels. The results revealed that subclinical variation symptoms of ASD were associated with less stable local dynamics, independent of task context. Further, exploratory analyses suggested that instructions to ignore the distractor were associated with changes to local dynamics for symptoms of SAD but global dynamics for symptoms of ASD. Taken together, these findings highlight how individual differences in psychological factors can shape the dynamics of everyday behaviour in context-dependent ways.

## Introduction

The fluency of everyday behaviour masks remarkable complexity. The challenge underlying effective action is imposing – every movement comprises myriad components that must be tightly coordinated to be efficient, yet sufficiently adaptable to cope with countless contingencies (Bernstein, 1967; Turvey, 1990). People resolve this challenge in distinctive ways specific to their own characteristics, both physical (e.g., height, flexibility) and, central to the current research, psychological (e.g., personality, mood). Understanding everyday behaviour, therefore, requires capturing the impact of pertinent individual differences in the context of naturalistic activity. Here, we do so by considering the influence of variation in mental health symptoms through the lens of dynamical systems theory (Warren, 2006). As a key driver of wellbeing, recent research shows that subclinical variation in mental health impacts *inter*personal dynamics (e.g., Macpherson & Miles, 2023). Yet considerably less focus has been given to these effects at the *intra*personal level, particularly in the context of naturalistic activity. To this end, we pose a novel question: does variation in individual-level psychological characteristics – specifically symptoms associated with social anxiety or autism spectrum disorders (ASDs) – change the dynamics of everyday behaviour?

Complex adaptive behaviour emerges from the interaction of the individual, task, and environmental constraints that shape coordinated action (Newell, 1986; Warren, 2006). To date, much of the empirical work exploring the dynamical corollaries of mental health has been confined to contrived experimental settings (e.g., Varlet et al., 2014) or idiosyncratic therapeutic contexts (e.g., Wiltshire et al., 2020). While these approaches offer high levels of experimental control and clinical relevance respectively, they raise concerns about representative design (Brunswik, 1956). Specifically, the extent to which the constraints governing behaviour in laboratory or clinical settings represent those operating in more everyday contexts is unclear. Capturing behavioural dynamics in the ‘wild’, that is, within the context of naturalistic everyday activity (Hutchins, 1995) is a key step in extending this line of research. Here, we do so by unobtrusively quantifying gait dynamics while participants walked a pre-set path during a regular day on a university campus.

### Context, Mental Health, and the Dynamics of Everyday Behaviour

Although primarily drawn from studies conducted in laboratory or therapeutic settings, there are empirical grounds to anticipate associations between symptoms of social anxiety disorder (SAD) or ASD and the dynamics of everyday behaviour. In brief, individuals diagnosed with these disorders exhibit idiosyncratic movement patterns (e.g., Feldman et al., 2019; Lum et al., 2020). For SAD, changes to the magnitude and variability of behaviour are reliably documented (e.g., Geh et al., 2011; Gong et al., 2019), while in ASD individuals often show a restricted, but more intrapersonally variable, range of motion (e.g., Kindregan et al., 2015; Weiss et al., 2013). Indeed, it has been speculated that movement differences at the level of the individual may underlie the disruptions to interpersonal dynamics commonly associated with symptoms of SAD or ASD (Cook, 2016; Macpherson & Miles, 2023). Notably, such individual-level movement atypicalities are not exclusive to clinical diagnoses and can be observed at subclinical levels more broadly (Macpherson et al., 2020). This suggests that, consistent with continuum approaches to mental health, symptoms associated with SAD and ASD may be implicated in shaping daily routine activity across the population (Goldberg, 2000; Knappe et al., 2009).

Capturing such effects, however, demands consideration of context. Many mental health symptoms are context-dependent (Ouliaris, 2020; Wakefield & First, 2012). To illustrate, relevant contextual factors – such as a shift in conversation topic (Asher et al., 2020), a change in an observer’s attentional focus (Macpherson et al., 2024) or the potential for evaluation (Geh et al., 2011) – alter how symptoms of SAD manifest in movement. Yet, laboratory studies typically neutralise context in the interest of experimental control, while clinical settings come with therapy-specific contextual limitations (e.g., clinician-client roles). To date, no research has systematically manipulated contextual factors while examining movement differences related to SAD and ASD in everyday settings. Addressing this gap, the present study introduces a contextual manipulation in which participants perform a secondary task framed as either a recall test or an irrelevant distraction.

### Capturing the Multi-Scaled Dynamics of Everyday Behaviour

Alongside consideration of context, to appropriately characterise everyday activity, it is necessary to account for its non-linear, multi-scaled nature (Warren, 2006). Behaviour unfolds across interconnected timescales from moment-to-moment fluctuations to longer-term patterning over extended periods. Researchers, however, have typically concentrated on either the local, time-dependent attributes (e.g., the degree of movement stability, Riley et al., 1999) *or* the global, time-invariant qualities (e.g., the structure of behavioural variability, Stephen et al., 2008) of behaviour. Given that constraints on behaviour may manifest differently across these levels of analysis, here we consider both the local and global dynamics of gait as means to provide insight into distinct, yet complementary aspects of everyday activity (Coey et al., 2014).

Measures of local dynamics quantify the degree to which moment-to-moment fluctuations in behaviour exhibit stable, repetitive patterns. High levels of repetition indicate regular and predictable behaviour that is resistant to disruption, whereas lower levels reflect irregular, unstable, and more readily perturbed patterns of activity. Capturing dynamics at this scale can provide a window into the control processes that govern behaviour (Coey et al., 2014; Likens et al., 2015). Changes to local dynamics can reflect the short-term sensorimotor adjustments made when behaviour is perturbed. To this end, a wide range of physical and psychological factors, including ageing (e.g., Brick et al., 2018), vestibular disorder (e.g., Labini et al., 2011), cognition (e.g., Pellecchia & Shockley, 2005; Riley et al., 2005; Riley & Clark, 2003), and personality (e.g. Danvers et al., 2020), have been shown to shape local dynamics. Of relevance to the current research, changes to local level movement dynamics have recently been associated with subclinical variation in SAD, an effect that was limited to situations where performance was monitored by a peer (Macpherson & Miles, 2023).

By comparison, global measures of behavioural dynamics reference the variability that aggregates across local states and captures the time-invariant structure of movement. Adaptive activity is reflected in patterns of behavioural variability that exhibit self-similarity and long-range dependencies (Kello et al., 2010; Van Orden et al., 2011). Termed 1/*f* scaling (‘pink noise’), this pattern is argued to reflect a balance between flexibility and stability (Van Orden et al., 2003) that lies between variability that is random, unstructured, and malleable (‘white noise’), and persistent and strongly correlated fluctuations in behaviour (‘brown noise’) that are more resistant to change. When constraints on action change, the organisation of behaviour and, consequently, the structure of behavioural variability, shifts. Research indicates that involuntary sources of control (e.g., task constraints) typically shift the structure of variability toward randomness (e.g., Decker et al., 2013; Dotov et al., 2016; Kiefer et al., 2009; Washburn et al., 2015). In contrast, voluntary sources of control (e.g., individual-enacted constraints) tend to increase the rigidity of behavioural fluctuations (e.g., Likens et al., 2015; Wijnants et al., 2009). In terms of the current work, one previous study has demonstrated that ASD children who exhibit higher levels of restricted and repetitive behaviour (i.e., a symptom of ASD) also show more persistent movement patterns (Romero et al., 2018).

### Current Research

The current study asked to what extent does subclinical variation in symptoms of SAD and ASD influence gait dynamics in a naturalistic everyday context? As well as being an exemplar of everyday activity, the dynamics of gait are well-established (e.g., Hausdorff et al., 1995; Labini et al., 2011), and changes to gait have previously been associated with clinical diagnoses of both SAD (e.g., Orcioli-Silva et al., 2021) and ASD (e.g., Lum et al., 2021). Participants completed two walking trials, the first of which served as a baseline. On the second trial, participants were given an additional distractor task in the form of an auditory language tutorial. Adding attentional load in this way influences behavioural dynamics at both local and global levels (e.g., Decker et al., 2013; Kiefer et al., 2009; Pellecchia & Shockley, 2005; Riley & Clark, 2003), and when delivered via headphones resembles daily behaviour for pedestrians on many university campuses.

Importantly, instructions regarding the distractor were varied between participants. Those in the ‘warned’ condition were told to expect a recall test at the conclusion of the trial. This manipulation imposes explicit evaluative and processing demands on participants, effects that have direct relevance to symptoms of SAD (e.g., fear of evaluation; Chen et al., 2020; Clark & Wells, 1995) and ASD (e.g., executive functioning differences; Demetriou et al., 2018; Hill, 2004) respectively. In the ‘surprised’ condition, participants were told to ignore the distractor, thereby creating an ambiguous task context given the continuous presence of the audio conflicted with the experimenter’s instructions. Ambiguity of this kind has been shown to reinforce evaluative concern in social anxiety (Clark & McManus, 2002) and to challenge pragmatic inference in autism (Pijnacker et al., 2009; Vicente et al., 2024). Varying these instructions (i.e., warned vs. surprised recall) created systematic differences in task constraints that map directly to processes implicated in SAD and ASD.

### Predictions

Guided by the available literature, we initially outline hypotheses regarding the effects of the distraction task on gait dynamics at the local and global levels separately.

**H1.** Behaviour will be more locally stable when participants are distracted versus not (e.g., Pellecchia & Shockley, 2005; Riley & Clark, 2003), an effect that will be more pronounced when warned of the recall test (cf. surprised).
**H2.** The structure of global behavioural variability will be more random (i.e., ‘white’ noise) when participants are distracted versus not (e.g., Kiefer et al., 2009), an effect that will be more pronounced when warned of the recall test (cf. surprised).

Adopting an exploratory stance, we also speculate as to the effects of SAD/ASD symptoms on gait dynamics and how these may be influenced by the distraction task instructions. When considering the interaction between symptoms of SAD/ASD and distraction task instructions, while we note the ambiguity inherent to the surprised condition may present different constraints to those in the warned condition, we suspect any influence of the instructions to ignore the distractor task will be more subtle than when explicitly informed of the recall test. Specifically, we anticipate that:

**EH1.** Higher symptom levels will be associated with gait dynamics that are less stable at the local level (e.g., Macpherson & Miles, 2023), an effect that will be more pronounced when participants are warned of the recall test (cf. surprised).
**EH2.** Higher symptom levels will be associated with gait dynamics that are more persistent at the global level (e.g., Romero et al., 2018), an effect that will be more pronounced when participants are warned of the recall test (cf. surprised).

## Method

### Participants and Design

A power analysis was conducted to determine the sample size required to detect relationships between symptoms of SAD/ASD and measures of gait dynamics at the local and global levels. The analysis was run using SIMR (v 1.0.7; Green & Macleod, 2016), a software package in R (v 4.3.0; R Core Team, 2023), using 1,000 simulations. Anticipated effect sizes were informed by prior research that considered the influence of individual and task constraints on movement dynamics across both local and global levels of analysis (e.g., Douglas et al., 2022).^1^ The simulations indicated that *n* = 90 was sufficient to achieve at least 80% power for detecting these effects with a significance criterion of α = .05.

To ensure an adequate sample, 114 participants were recruited to take part in the study (78 self-identified as female, and 36 as male) who were aged between 18 and 50 years (*M* = 20.99 years; *SD* = 5.82 years). Of these, 95 were undergraduate students who took part in exchange for course credit, and 19 were community members who were reimbursed for their time (A$20). Only individuals aged 18 or over and with no known movement impairments were eligible to participate. The data from 19 participants were excluded due to weather conditions necessitating the use of an alternate path.^2^ Additionally, data from two participants were excluded due to technical issues with gait capture, resulting in a final sample of 93 participants (female = 64, male = 29; aged 18–50 years; *M* = 21.02 years; *SD* = 5.73 years).

The study employed a mixed design whereby each participant performed two walking trials in fixed order, the first with no distraction (i.e., baseline trial) and subsequently with a distraction (see Procedure for details of the distractor task). For the second trial, participants were randomly assigned to a recall test condition whereby either they were told to ignore the distractor (i.e., surprised condition, *n* = 43) or informed there would be a subsequent memory test (i.e., warned condition, *n* = 50). The study was reviewed and approved by the University of Western Australia Human Research Ethics Office (2022/ET000163).

### Procedure

Upon arrival to the laboratory participants, provided written consent and were asked to report their age and gender (free response format) and whether they had any condition or injury that interfered with their movement. No participants identified any movement issues. Participants then completed self-report versions of the Liebowitz Social Anxiety Scale (LSAS; Liebowitz, 1987) and the Autism Spectrum Quotient-short form (AQ-S; Hoekstra et al., 2011). Both scales have strong psychometric properties^3^ and are widely used to capture subclinical variation in traits associated with SAD and ASD (e.g., Macpherson & Miles, 2023). Summary statistics for the LSAS and AQ-S for the present sample are presented in Table 1.

**Table 1.** Summary Statistics for the LSAS and AQ-S.

Measure	LSAS	AQ-S
Range	4–125	41–88
Mean	59.22	64.51
Standard deviation	24.28	8.19
Skew	0.19	0.15
Kurtosis	−0.36	0.19

*Note.* LSAS – potential range = 0–144; AQ-S – potential range = 28–112.

Where participants neglected to respond to an item, the missing value was replaced with the mean of the relevant subscale. This resulted in the replacement of 8 missing values across the sample (i.e., <0.1% of answers).

AQ-S = Autism Spectrum Quotient-short form; LSAS = Liebowitz Social Anxiety Scale.

Next, the experimenter escorted the participant to the starting location of the walking trials. The walking path was an unobstructed straight stretch of paved surface on the university campus in an area with only moderate foot traffic. Once at the starting position, the participant was informed they would be completing two walking trials, first undistracted (i.e., not listening to anything^4^), followed by the same trial with the addition of an audio distractor played over headphones. Participants were required to walk for 250 m until they reached a designated turning point and then walk back to the starting position (i.e., 500 m total walking trial). They were told to walk at a comfortable pace and refrain from using their phone or interacting with other pedestrians. The participant was then fitted with an iPhone 7 to their right ankle using a lightweight ankle strap that captured their gait via the onboard accelerometer (sample rate = 100 Hz) and the MATLAB Mobile iOS application.

For the second (i.e., distracted) walking trial, participants were told that they would hear an English to Macedonian language tutorial, and they should either: ‘ignore the audio as much as possible and just complete the walk as you did previously’ (i.e., surprised condition) or ‘pay close attention to the audio as you will be tested on the content after completing the walk’ (i.e., warned condition). The distractor task comprised a pre-recorded series of 25 English-Macedonian word pairings (i.e., 50 words total) spoken aloud by a female fluent in both languages (e.g., mirror – *glédalo*, pencil – *móliv*; see Table S1 for the full list). The track began with a beep that indicated to the participant to start walking, followed by the word pairs presented every 4 s, (i.e., full track length = 200 s). The track was continuously looped to ensure participants were distracted for the full duration of the walking trial (i.e., the recording didn’t end prematurely due to differences in walking speed).

Following the completion of the second walking trial, the participant’s recall of the language tutorial (i.e., distraction task) was assessed as a manipulation check.^5^ Participants were also asked whether they spoke languages other than English to ensure no cross-over with Macedonian. No participants reported fluency in related languages. They were then debriefed and dismissed. Each testing session last approximately 45 min.

### Data Pre-Processing and Estimation of Dynamics

#### Pre-Processing

Acceleration data were recorded at 100 Hz along the *x*, *y*, and *z* axes.^6^ To prepare for analysis, a single magnitude vector was computed and filtered using a fourth-order low-pass Butterworth filter (10 Hz cut-off). Non-walking periods at the start and end of each trial were excluded by identifying the first and last step. To do so, initially, steps were detected as local maxima in the acceleration timeseries exceeding 0.5 SD above the median with a minimum separation of 70 samples. Each timeseries was then visually inspected and adjustments made to the SD threshold as necessary. Turn-around points were removed by excluding samples where the interval between consecutive local maxima exceeded ±3 SD from the median (Hausdorff et al., 1997, 2001). Again, each timeseries was visually inspected, and SD cut-offs adjusted as needed. The processed data were then analysed using Recurrence Quantification Analysis (RQA; Zbilut & Webber, 1992) to assess local movement dynamics, and Detrended Fluctuation Analysis (DFA; Peng et al., 1994) to quantify global movement dynamics.

#### Recurrence Quantification Analysis

RQA was employed to estimate local level (i.e., moment-to-moment fluctuations) in gait dynamics across three complementary indices: recurrence (%REC), determinism (%DET) and maxline (MaxL),^7^ that have previously been employed in related work (e.g., Macpherson & Miles, 2023; Romero et al., 2016). As a measure of recurrent activity (Richardson et al., 2007; Shockley et al., 2003), %REC reflects the extent to which patterns of acceleration magnitude are revisited over time, thereby indexing the consistency of gait fluctuations. %DET quantifies the extent to which these recurrent states form regular or predictable sequences (Curtin et al., 2017; McCamley et al., 2017), indicating the degree to which changes in acceleration magnitude unfold in ordered or structured ways. Finally, MaxL captures the maximum duration over which stable acceleration magnitude patterns remain unchanged, thereby indexing the stability of gait (Richardson et al., 2007; Shockley et al., 2003). For the current study, MaxL was normalised with respect to the length of each time series (i.e., proportional maxline; pMaxL). Across all metrics, higher values are indicative of more coordinated, regular, and stable patterns of gait dynamics.

As per standard protocols (e.g., Coey et al., 2014), the delay (14) and embedding dimension (9) were estimated using the first minimum of the average mutual information and first minimum of the false nearest neighbour analysis, respectively. To maintain an average recurrence rate of below 5%, the radius was set to 15 (Richardson et al., 2007; Shockley et al., 2003). These parameter values were applied globally across all participants.

#### Detrended Fluctuation Analysis

DFA was employed to estimate the global level (i.e., time-invariant structure) gait dynamics. This approach quantifies long-range temporal correlations in time series data by measuring how fluctuations scale across various window sizes (e.g., 8, 16, 64. . . data points). The resultant scaling exponent (α) provides an index of the structure of the time series whereby α ≈ .5 corresponds to random, unstructured variability (i.e., white noise), α ≈ 1 corresponds to 1/*f* scaling or fractal variability (i.e., pink noise), and α ≈ 1.5 corresponds to a highly correlated pattern of variability (i.e., Brown noise).

In this way, RQA and DFA were used to capture complementary (i.e., local and global) levels of gait dynamics. Although RQA indices are computed across the full recurrence plot, they reflect the local properties of gait by quantifying the extent to which short-term fluctuations revisit similar states (e.g., %REC, %DET, pMaxL). These metrics index moment-to-moment regularity and resistance to perturbation rather than the long-range correlation structure of the timeseries. In contrast, DFA characterises the global structure of variability by assessing whether fluctuations exhibit scale-free dependencies across timescales. Taken together, RQA captures the local regularity of ongoing behaviour, whereas DFA assesses the global, time-invariant organisation of variability.

#### Statistical Analyses

To evaluate the hypotheses, separate linear mixed effects models were constructed for each behavioural metric (i.e., %REC, %DET, pMaxL, α), using the lme4 (Bates et al., 2015) and lmerTest (Kuznetsova et al., 2017) packages in R (v 4.1.3; R Core Team, 2023). All continuous predictors and dependent variables were centred and scaled (*z*-scored) prior to inclusion in the models, ensuring that the resulting regression coefficients reflect standardised effects. As a preliminary test of the effect of the audio distraction, we initially constructed models that examined the effect of the distraction task (coded as: 0 = no distraction, 1 = distraction). Next, we constructed models that examined the effect of recall condition (coded as: 0 = no distraction, 1 = warned, 2 = surprised).^8^ Results tables for these models can be found in the supplementary materials. Finally, we considered the full experimental design with models that specified fixed effects of recall condition (coded as above) and one of the questionnaire measures (i.e., LSAS for SAD symptoms; AQ-S for ASD symptoms). Degrees of freedom and *p*-values were calculated using the Satterthwaite method of approximation included in the lmerTest package, and the random effects structure comprised a by-participant random intercept. This is the maximal model that would converge (Barr et al., 2013).^9^ Interaction effects were decomposed by estimating Tukey-corrected pairwise comparisons using the emmeans package (Lenth, 2021). For dependent variables that were not normally distributed, an appropriate transformation was applied.^10^ The dataset and R code used to compute the models are available on the Open Science Framework (https://osf.io/yz429/).

When interpreting results and evaluating the exploratory hypotheses, we adopted a discovery-oriented approach (Tong, 2019; Tukey, 1977) whereby we considered patterns in the data that offered potential insight into the effects of interest even if they did not meet conventional thresholds for statistical significance (*p* < .05). This perspective aligns with critiques of overreliance on null hypothesis significance testing (e.g., De Groot, 1956/2014; Wagenmakers et al., 2012) and acknowledges ongoing debates surrounding the *p*-value estimation in linear mixed models (e.g., Kuznetsova et al., 2017). When adopting this stance, however, we are mindful of the need to exercise caution when interpreting findings.

## Results

### Behavioural Dynamics and Distraction

At the local level (H1), participants’ gait dynamics revealed a trend towards higher levels of %REC (i.e., more regular gait) in the distraction compared to the no distraction condition (β = .16, *SE* = 0.09, *t*(92) = 1.74, *p* = .09). No effects were observed for %DET (β = .04, *SE* = 0.06, *t*(92) = 0.64, *p* = .53), or pMaxL (β = −.18, *SE* = 0.12, *t*(92) = −1.53, *p* = .13). At the global level (H2), α was significantly lower (i.e., less structured) in the distraction compared to the no distraction condition (β = −0.26, *SE* = 0.10, *t*(92) = −2.60, *p* = .01). Results are displayed in Table S2.

### Behavioural Dynamics and Recall Instructions

**Figure 1.**
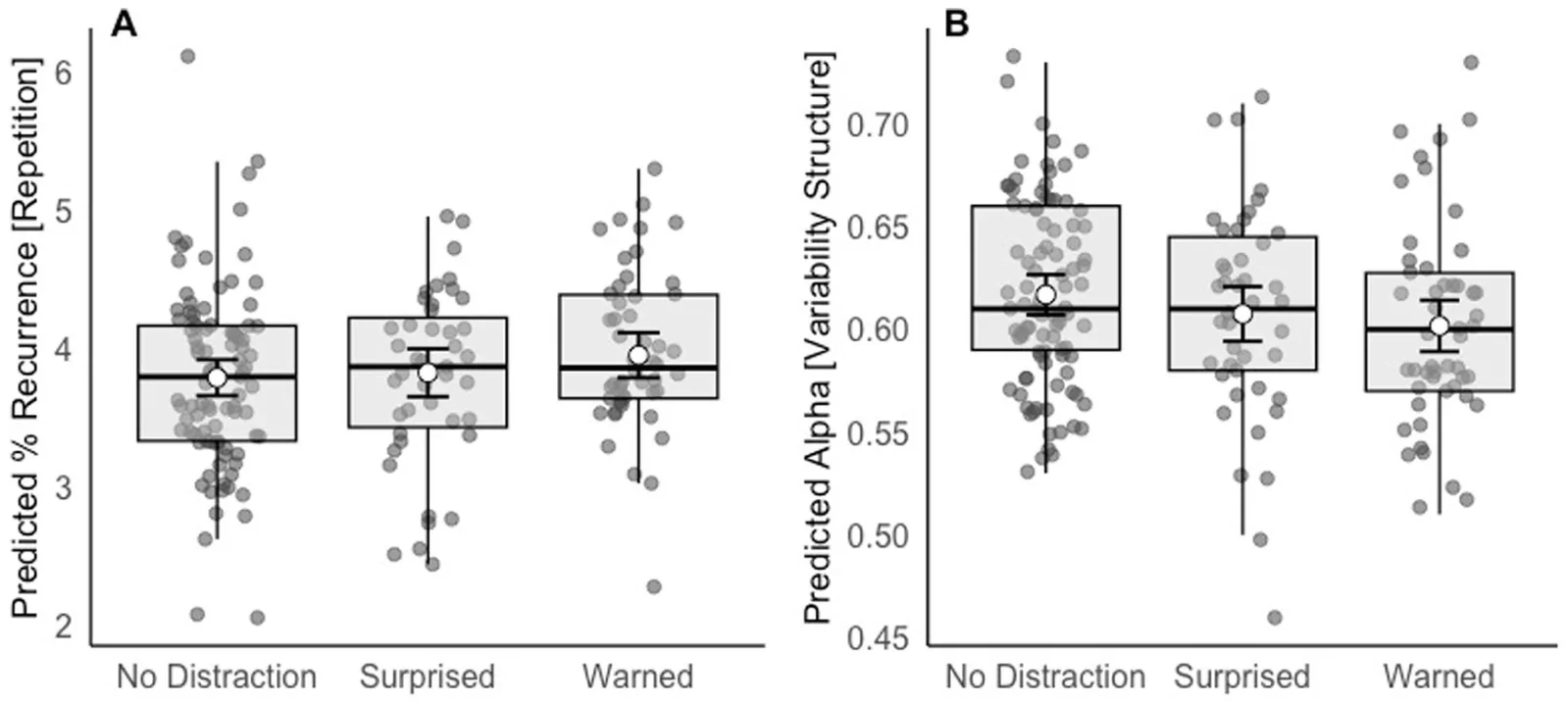
The effect of recall instructions on %REC (Panel A) and α (Panel B). *Note*. The box depicts the median and the IQR of the raw data. Whiskers extend to the minimum and maximum values within ±1.5 × IQR from the upper and lower quartiles excluding outliers. The white circle indicates the predicted mean derived from the relevant model with error bars representing 95% confidence intervals. Raw data points for each participant are plotted for clarity. IQR = interquartile range.

At the local level (H1), pairwise comparisons between recall conditions (i.e., no distraction/surprised/warned; see Tables S3 and S4) revealed a trend towards an increase in %REC in the warned compared to no distraction condition, (β = −.25, *SE* = 0.12, *t*(108) = −2.09, *p* = .10). There were no significant differences in %REC between the no distraction and surprised (β = −.06, *SE* = 0.13, *t*(111) = −0.44, *p* = .90), or warned and surprised (β = .20, *SE* = 0.17, *t*(136) = 1.18, *p* = .47; Figure 1, panel A) conditions. No significant effects were found for %DET or pMaxL.

At the global level (H2), pairwise comparisons revealed a significant decrease in α in the warned compared to no distraction condition, indicating a shift toward more random patterns of movement variability (β = .32, *SE* = 0.13, *t*(110) = 2.45, *p* = .04). There were no significant differences in α between the no distraction and surprised (β = .19, *SE* = 0.14, *t*(114) = 1.42, *p* = .34) or the warned and surprised (β = −.12, *SE* = 0.18, *t*(142) = −0.69, *p* = .77; Figure 1, panel B) conditions.

### Behavioural Dynamics and Mental Health

**Figure 2.**
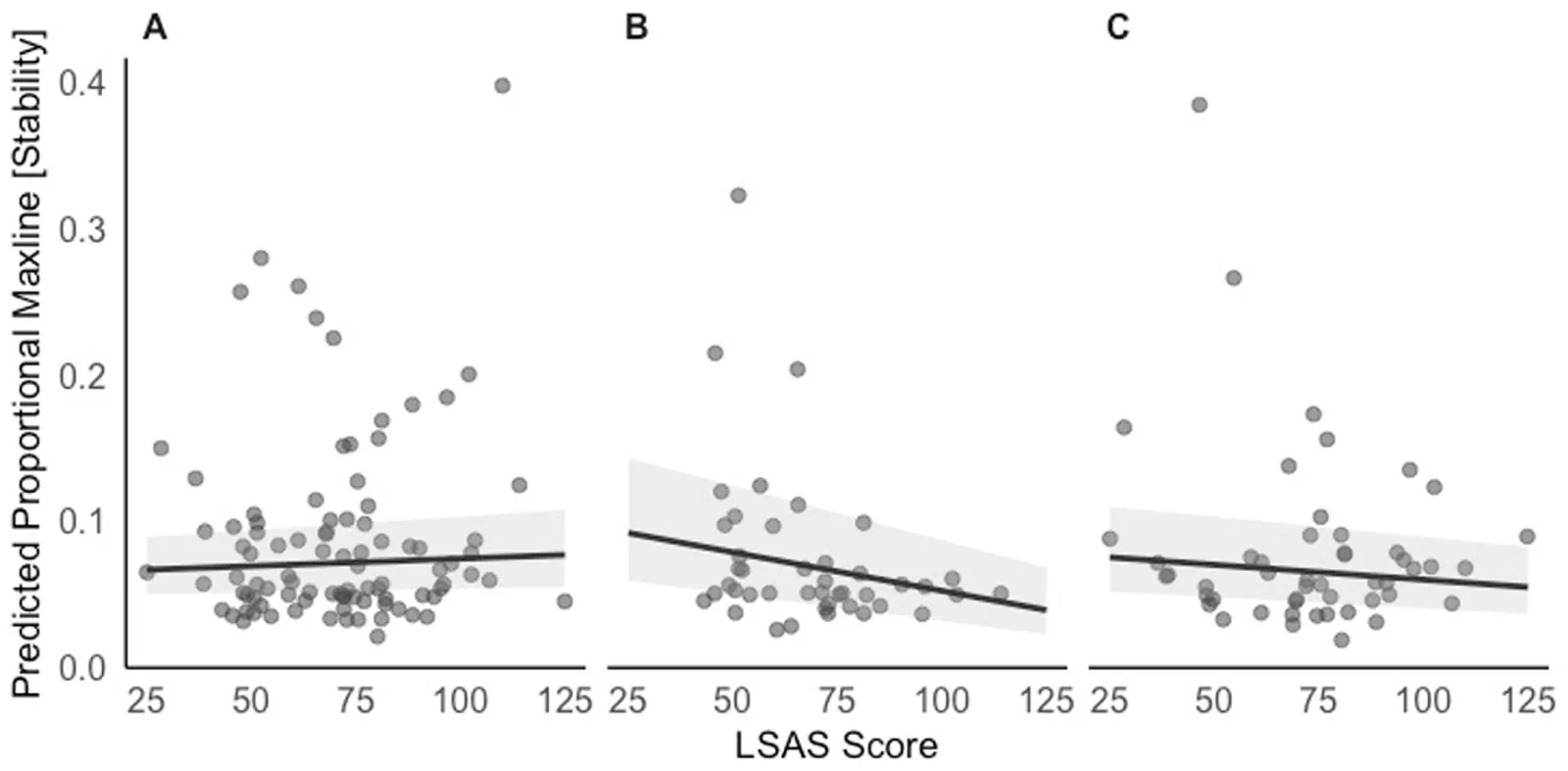
The relationship between LSAS score and pMaxL for each condition. Panel (A) represents the no distraction condition, Panel (B) the surprised condition and Panel (C) the warned condition. *Note*. The raw (untransformed) data points for each participant are plotted alongside the model’s linear predictions for clarity. The shaded area represents 95% CI’s for the linear predictions. LSAS = Liebowitz Social Anxiety Scale.

There were no significant main effects of symptoms of SAD (i.e., LSAS scores) on local or global gait dynamics (EH1, EH2), nor any significant relationships between LSAS scores and gait dynamics across conditions (see Tables 2 and 3). However, there was a trend when considering effects on pMaxL. Inspection of the slopes revealed a marginal negative relationship with LSAS in the surprised condition (β = −.30, *SE* = 0.17, *t*(179) = −1.80, *p* = .07; Figure 2, panel B). This suggests that higher LSAS scores may be associated with decreased local movement stability when individuals are instructed to ignore a distraction. No equivalent pattern was present in the no distraction (β = .05, *SE* = 0.10, *t*(163) = 0.49, *p* = .63; Figure 2, panel A) or warned conditions (β = −.11, *SE* = 0.13, *t*(178) = −0.87, *p* = .38; Figure 2, panel C).

**Table 2.** Summary of the Estimated Marginal Trends From Models Examining the Influence of Condition and LSAS/AQ-S on Each Metric of Behavioural Dynamics (i.e., %REC, %DET, pMaxL, α).

Dependent variable	Condition	*B*	*SE*	*df*	95% CI	*t*	*P*
LSAS							
%REC	No distraction	−0.009	0.104	132	[−0.215, 0.197]	−0.083	.934
	Warned	−0.092	0.123	168	[−0.334, 0.150]	−0.753	.453
	Surprised	−0.173	0.153	180	[−0.475, 0.129]	−1.129	.260
%DET	No distraction	0.001	0.105	109	[−0.208, 0.209]	0.005	.996
	Warned	−0.020	0.115	139	[−0.248, 0.207]	−0.175	.861
	Surprised	−0.041	0.133	173	[−0.303, 0.222]	−0.305	.761
pMaxL	No distraction	0.051	0.104	163	[−0.154, 0.255]	0.488	.627
	Warned	−0.112	0.128	178	[−0.364, 0.141]	−0.873	.384
	Surprised	−0.301	0.167	179	[−0.630, 0.028]	−1.804	.073
Alpha (α)	No distraction	0.069	0.103	142	[−0.134, 0.274]	0.670	.504
	Warned	0.102	0.124	173	[−0.142, 0.374]	0.826	.410
	Surprised	−0.106	0.157	179	[−0.415, 0.204]	−0.672	.502
AQ-S							
%REC	No distraction	−0.179	0.103	136	[−0.382, 0.024]	−1.746	.083
	Warned	−0.115	0.122	171	[−0.356, 0.127]	−0.938	.350
	Surprised	−0.272	0.148	180	[−0.356, 0.020]	−1.841	.067
%DET	No distraction	−0.222	0.103	109	[−0.426, −0.018]	−2.151	.034
	Warned	−0.112	0.114	142	[−0.337, 0.112]	−0.989	.325
	Surprised	−0.195	0.128	171	[−0.448, 0.057]	−1.527	.129
pMaxL	No distraction	−0.202	0.103	165	[−0.405, 0.001]	−1.967	.051
	Warned	−0.221	0.129	179	[−0.476, 0.033]	−1.714	.088
	Surprised	−0.141	0.161	179	[−0.458, 0.177]	−0.875	.383
Alpha (α)	No distraction	−0.027	0.103	138	[−0.232, 0.177]	−0.265	.791
	Warned	−0.190	0.124	172	[−0.435, 0.055]	−1.532	.127
	Surprised	0.260	0.150	180	[−0.036, 0.556]	1.732	.085

*Note*. AQ-S = Autism Spectrum Quotient-short form; LSAS = Liebowitz Social Anxiety Scale.

**Table 3.** Summary of the Pairwise Comparisons From Models Examining the Influence of Condition and LSAS/AQ-S on Each Metric of Behavioural Dynamics (i.e., %REC, %DET, pMaxL, α).

Contrast	*B*	*SE*	*df*	95% CI	*T*	*p*
LSAS						
No distraction – warned	0.084	0.114	99.9	[−0.187, 0.355]	0.734	.744
No distraction – surprised	0.164	0.146	111.4	[−0.183, 0.512]	1.123	.502
Warned – surprised	0.081	0.175	130.8	[−0.335, 0.496]	0.459	.891
No distraction – warned	0.021	0.079	94.8	[−0.167, 0.209]	0.262	.963
No distraction – surprised	0.041	0.103	99.6	[−0.204, 0.286]	0.398	.916
Warned – surprised	0.020	0.127	106.8	[−0.282, 0.322]	0.160	.986
No distraction – warned	0.162	0.142	108	[−0.174, 0.499]	1.146	.488
No distraction – surprised	0.351	0.177	128	[−0.069, 0.772]	1.981	.121
Warned – surprised	0.189	0.205	162	[−0.295, 0.673]	0.924	.626
No distraction – warned	−0.033	0.122	103	[−0.323, 0.257]	−0.270	.961
No distraction – surprised	0.175	0.156	117	[−0.195, 0.544]	1.123	.502
Warned – surprised	0.208	0.185	141	[−0.230 0.646]	1.124	.501
AQ-S						
No distraction – warned	−0.064	0.116	103	[−0.339, 0.211]	−0.557	.843
No distraction – surprised	0.092	0.142	113	[−0.245, 0.429]	0.650	.793
Warned – surprised	0.157	0.172	134	[−0.252, 0.565]	0.909	.636
No distraction – warned	−0.110	0.079	95.6	[−0.298, 0.079]	−1.383	.354
No distraction – surprised	−0.026	0.099	99.7	[−0.261, 0.208]	−0.268	.961
Warned – surprised	0.083	0.124	107.6	[−0.211, 0.377]	0.673	.780
No distraction –warned	0.019	0.144	109	[−0.324, 0.362]	0.130	.991
No distraction – surprised	−0.062	0.174	126	[−0.473, 0.350]	−0.355	.933
Warned – surprised	−0.080	0.202	165	[−0.557, 0.396]	−0.399	.916
No distraction – warned	0.163	0.119	104	[−0.121, 0.446]	1.365	.363
No distraction – surprised	−0.287	0.146	114	[−0.634, 0.059]	−1.968	.125
Warned – surprised	−0.450	0.177	137	[−0.869, 0.031]	−2.544	.032

*Note*. AQ-S = Autism Spectrum Quotient-short form; LSAS = Liebowitz Social Anxiety Scale.

**Figure 3.**
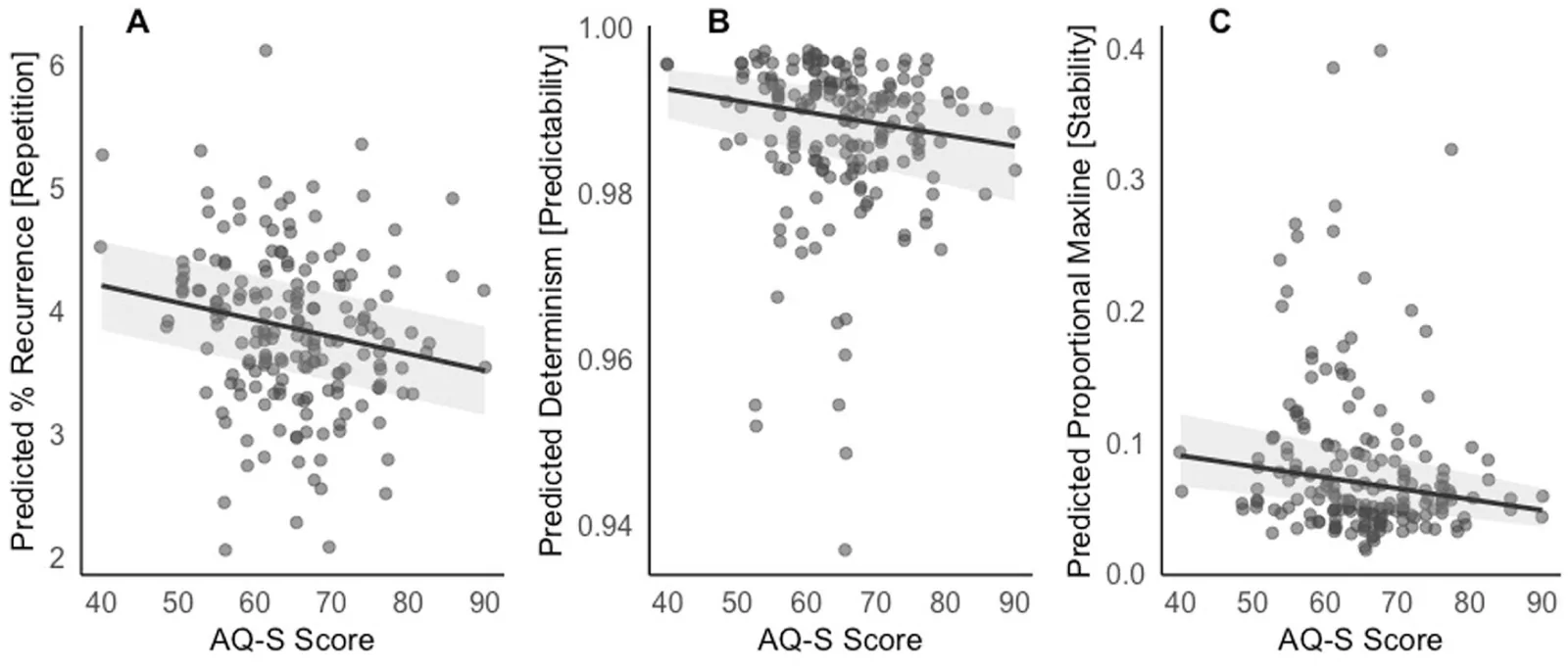
The relationship between AQ-S score and %REC (Panel A), %DET (Panel B) and pMaxL (Panel C). *Note*. The raw (untransformed) data points for each participant are plotted alongside the model’s linear predictions for clarity. The shaded area represents 95% CI’s for the linear predictions. AQ-S = Autism Spectrum Quotient-short form.

Notably, AQ-S scores (i.e., symptoms of ASD) showed consistent negative associations with local gait dynamics (EH1), %REC (β = −.19, *SE* = 0.09, *t*(98.8) = −2.02, *p* = .05; Figure 3, panel A), %DET (β = −.18, *SE* = 0.09, *t*(94.5) = −1.78, *p* = .08; Figure 3, panel B), and pMaxL (β = −.19, *SE* = 0.09, *t*(104) = −2.17, *p* = .03; Figure 3, panel C). These results indicate that higher AQ-S scores were associated with less regular local gait dynamics. Pairwise comparisons of the relationship between AQ-S and each local metric across conditions, however, revealed no differences, indicating that for ASD, local-level dynamics were influenced independent of task context.

**Figure 4.**
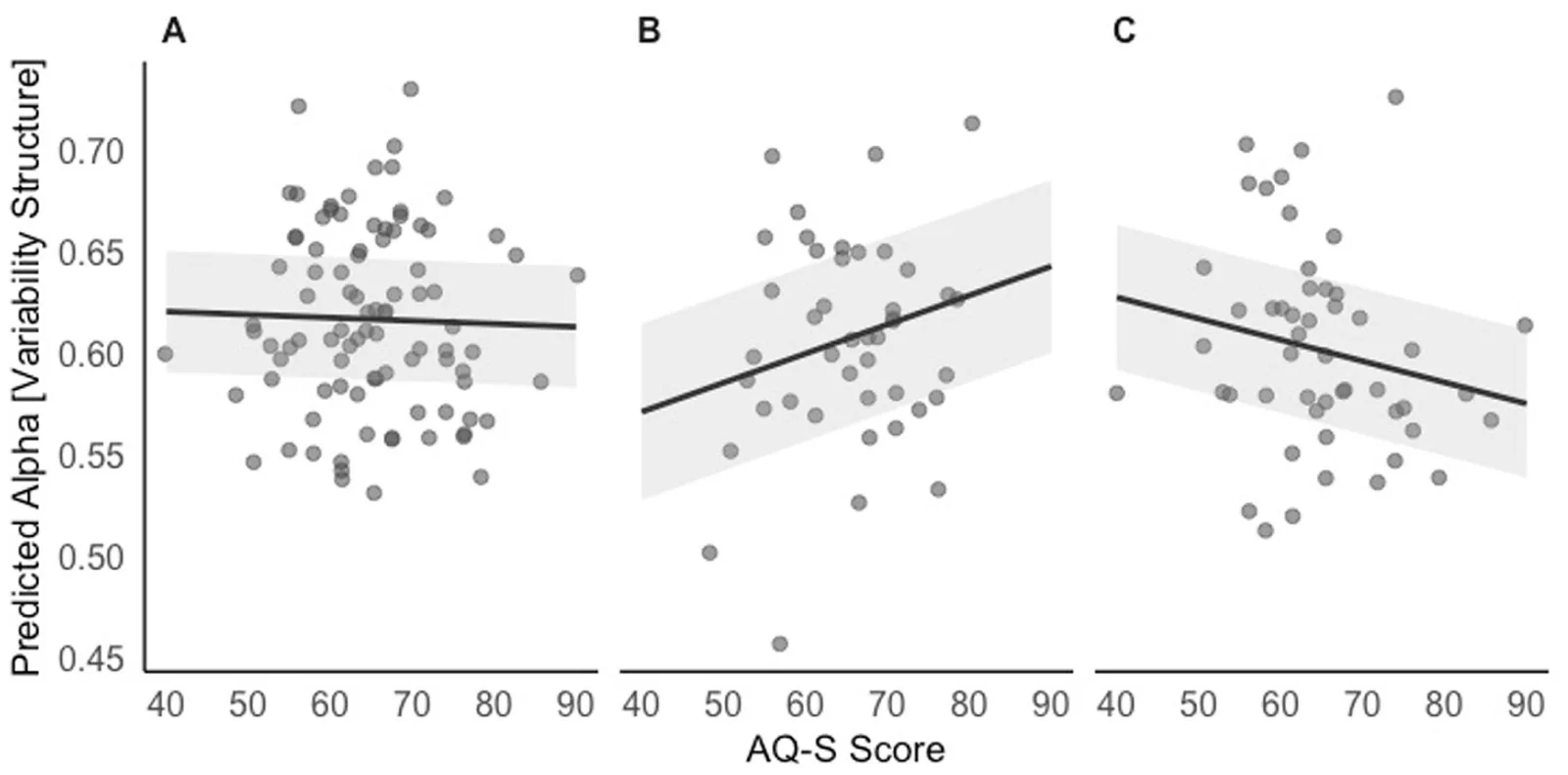
The relationship between AQ-S score and α for each condition. Panel (A) represents the no distraction condition, Panel (B) the surprised condition and Panel (C) the warned condition. *Note*. The raw data points for each participant are plotted alongside the model’s predictions for clarity. The shaded area represents 95% CI’s for the linear predictions. AQ-S = Autism Spectrum Quotient-short form.

At the global level, there was no main effect of AQ-S scores on α (EH2). However, pairwise comparisons indicated a significant difference in the relationship between AQ-S and α between the surprised and warned conditions (β = −.45, *SE* = 0.18, *t*(137) = −2.54, *p* = .03). Inspection of the slopes revealed a marginal positive relationship between AQ-S and α in the surprised condition (β = .26, *SE* = 0.15, *t*(180) = 1.73, *p* = .09; Figure 4, panel B), indicating that AQ may be associated with more persistent global behavioural variability when participants were told to ignore the distractor. No effects were present in the no distraction (β = −.03, *SE* = 0.10, *t*(138) = −0.27, *p* = .79; Figure 4, panel A) or warned conditions (β = −.19, *SE* = 0.12, *t*(172) = −1.53, *p* = .13; Figure 4, panel C).

## Discussion

The current study explored the extent to which typical (i.e., subclinical) variation in symptoms of SAD and ASD impacts the dynamics of everyday behaviour. Acknowledging the context-dependency of mental health symptomology, we varied contextually relevant task constraints via a secondary distractor while participants performed a routine activity – walking on a university campus.

### Behavioural Dynamics and Distraction

Consistent with existing laboratory research (e.g., Decker et al., 2013; Kiefer et al., 2009; Pellecchia & Shockley, 2005; Riley & Clark, 2003), the presence of the distraction task was seen to influence the dynamics of behaviour. While the anticipated pattern of increased local gait stability when distracted was evident, these effects did not reach statistical significance and therefore offer only tentative support for H1. When considering the global dynamics, however, there was clear support for H2 – the structure of behavioural variability was more random when participants were distracted, an effect that appeared to be driven by knowledge of the pending recall test. Increased cognitive load was therefore seen to influence gait dynamics outside the controlled confines of the laboratory.

### Behavioural Dynamics and Mental Health

The results also provided partial support for EH1. While there was no evidence of a bivariate relationship between symptoms of SAD and gait, symptoms of ASD showed consistent negative associations with indices of local-level dynamics. Specifically, participants reporting higher ASD symptom levels exhibited patterns of gait acceleration whereby short-term (i.e., moment-to-moment) fluctuations were repeated with less regularity, indicative of disruption to local dynamics (e.g., locally less repetitive, predictable, and stable gait). Notably, similar gait patterns have been observed in laboratory studies with ASD populations (e.g., Lum et al., 2021; Rinehart et al., 2006; Weiss et al., 2013). Although this previous work has tended to focus on measures of discrete aspects of gait (e.g., stride length, stance width) rather than the dynamical properties, the data point to general patterns of increased variability/reduced regularity of gait parameters that vary with symptom severity within clinical populations (Biffi et al., 2018; Kindregan et al., 2015). While the current results are consistent with this work, it is important that future research examine clinical populations in more everyday situations before drawing any definitive conclusions regarding the parallels between the subclinical and clinical domains. Finally, there were no equivalent effects of SAD or ASD symptoms at the global level, thereby providing no support for EH2.

### Behavioural Dynamics, Distraction, and Mental Health

With respect to the exploratory predictions regarding the interaction of SAD/ASD symptoms and recall instruction, we found no support for the prediction that the influence of SAD/ASD would be most prominent in the warned condition. There were, however, two clear trends that were not anticipated and warrant consideration. For both SAD and ASD, the data suggest associations with gait dynamics specific to the surprised condition. For SAD, the stability of local gait dynamics showed a marginal negative association with symptom levels in the surprised condition. On the other hand, there was a trend towards an association between symptoms of ASD and more persistent global gait patterns when participants were told to ignore the distractor. Interestingly, those warned of the recall test showed a contrasting pattern (i.e., increased random variability as a function of more symptoms), although this did not reach significance. These results underscore the context-dependency inherent to symptoms of mental health (Ouliaris, 2020; Wakefield & First, 2012).

As noted, these effects were contrary to the expectation that knowledge of the recall test would amplify the impact of the audio distraction. This raises the possibility that factors beyond any direct effect of increased cognitive load may be at play. For SAD, we speculate that the ambiguity of the instruction (i.e., being given something to listen to, but told to ignore it) may have heightened feelings of uncertainty or concern about evaluation (Clark & McManus, 2002; Stopa & Clark, 1993) and provided additional evaluative threat compared to a predictable recall test (warned condition). In terms of ASD, Pijnacker et al. (2009) report evidence that ASD adults can experience challenges with pragmatic inference, often leading to increases in literal thinking (Vicente et al., 2024). As a result, participants with higher levels of ASD traits may have been more likely to successfully disregard the distractor audio as instructed. This interpretation has some support from patterns of performance in the recall tests whereby there were trends towards ASD symptoms being associated with poorer recall in the surprised but not the warned condition.^11^ Taken together, these explanations provide a plausible account for why symptoms of SAD and ASD were more impactful in the surprised condition. However, they raise an associated question of why the warned condition did not amplify the effect of symptoms as initially predicted. One possibility is that providing explicit notice of the upcoming recall tests may have reduced uncertainty and allowed participants to organise their behaviour around a predictable and stable task goal. In turn, this may have attenuated the symptom-related differences that emerged in the more ambiguous surprised condition. However, these interpretations are speculative and, without additional research, should be treated judiciously.

The present data also highlighted a potential difference in the way SAD and ASD impact gait dynamics. Symptoms of SAD showed a trend towards a context-dependent (i.e., surprised condition) influence on local gait dynamics. For symptoms of ASD, however, there was also evidence of context-dependent impact, but to global gait properties. We suggest this is a preliminary indication that real-time gait adaptations may be modulated by different psychological factors than the longer-range variability structures that characterise the time-invariant properties of behaviour. In more broad theoretical terms, contrasts between effects on dynamics at the local vs. global level can reflect distinctions in underlying behavioural control processes (Coey et al., 2014; Likens et al., 2015). One possibility is that symptoms of SAD may impact local-level gait dynamics through heightened evaluative self-monitoring and increased vigilance to the environment (Mogg & Bradley, 1998). These behaviours involve rapid fluctuations in attentional processes that may also be revealed in short-term sensorimotor adjustments, such as the reduction in gait stability observed in the surprised recall condition. By contrast, symptoms associated with ASD (e.g., differences in executive function, cognitive rigidity, sensory-motor integration; Fournier et al., 2010, Hill, 2004, Lage et al., 2024) reflect more enduring tendencies that may be more evident in changes to the global organisation of behaviour. Although this interpretation offers novel insight into a meaningful contrast between local and global behavioural dynamics, the supporting evidence relies on statistically marginal interactions. Accordingly, the explanation we propose should be regarded as exploratory and hypothesis-generating rather than definitive evidence of dissociable processes relevant to symptoms of SAD/ASD. To progress this proposition, future research should systematically manipulate sources of gait perturbance (i.e., environmental, task, or individual constraints) in ways that demand more discrete (e.g., avoiding a pedestrian) versus enduring (e.g., challenging terrain) accommodation.

These findings extend beyond gait dynamics to illustrate how symptoms of psychopathology are expressed in embodied processes. From an embodiment perspective (e.g., Chemero, 2009; Wilson, 2002) rather than being confined to disembodied internal representations, psychological states constrain the organisation of ongoing behaviour, consistent with the fluctuations in gait observed here. Furthermore, when considered from the standpoint of dynamical systems theory (Warren, 2006), behaviour is seen to unfold across different temporal scales, and our results suggest that different aspects of psychopathology may constrain behavioural control processes at distinct timescales (i.e., local stability vs. global variability). This interpretation is also consistent with transdiagnostic frameworks that conceptualise psychopathology as emerging from dynamic networks of symptoms and processes, rather than discrete diagnostic categories (Borsboom et al., 2022; Roefs et al., 2022). We advocate, therefore, for future work to explore related domains of psychopathology, particularly disorders where disruption to motor behaviour has been noted (e.g., ADHD, Basic et al., 2024; MDD, Wüthrich et al., 2022) from an embodied perspective. By identifying commonalities and distinctions in the dynamical organisation of behaviour between conditions, this approach can help inform transdiagnostic perspectives of psychopathology (e.g., Kotov et al., 2017).

It should be noted that while representing a prototypical everyday activity, gait dynamics are inherently stable (McMahon, 1984). Given recent evidence to suggest that coordination stability may act as a boundary condition on the relationship between psychopathology and interpersonal dynamics (i.e., stable interpersonal coordination is more resistant to perturbation from individual differences, Macpherson & Miles, 2023; Macpherson et al., 2024), it is possible that gait may be more robust to the effects of individual difference factors than other, less stable, activities. Future research may look to extend the current work by employing naturalistic everyday behaviours that are dynamically more variable (e.g., preparing a meal) and therefore more open to perturbation via psychological factors. A limitation stemming from the study design also warrants noting. Due to the limited number of observations per participant, analyses were restricted to random-intercept only models (i.e., the maximally converging model structure). While this approach allows symptom effects to vary across conditions, it also assumes that within each condition, the relationship between symptoms and gait dynamics is consistent for all participants. Consequently, individual variability in these slopes cannot be estimated. Given such simplification may restrict detection of complex interactions, future work should employ experimental designs that support more flexible random-effects structures (e.g., more repeated observations, increased sample size) to better capture individual differences in these relationships.

## Conclusion

Taken together, the current results provide an important extension to the contemporary literature on behavioural dynamics. Individual differences in symptoms of psychopathology were seen here to constrain both the local and global dynamics of everyday naturalistic activity. While idiosyncratic movement patterns have previously been associated with both SAD and ASD (e.g., Feldman et al., 2019; Lum et al., 2021), to our knowledge, this is the first demonstration of such effects in a naturalistic setting within a typical (i.e., subclinical) population. By bridging the generalisability gap between laboratory and clinical settings and everyday behaviour (Brunswik, 1956), the current work underscores the complex multi-scaled nature of everyday behaviour and demonstrates that psychological factors shape naturalistic activity in contextually meaningful ways.

## Supplemental Material

sj-docx-1-qjp-10.1177_17470218261424186 – Supplemental material for Subclinical Variation in Mental Health Shapes the Dynamics of Everyday Behaviour Supplemental material, sj-docx-1-qjp-10.1177_17470218261424186 for Subclinical Variation in Mental Health Shapes the Dynamics of Everyday Behaviour by Amber J. Brown, Margaret C. Macpherson and Lynden K. Miles in Quarterly Journal of Experimental Psychology

## Notes

1. We took a conservative position whereby we used estimated effect sizes of each factor on α (global dynamics), on the basis that effects on local dynamics are anticipated to be larger. For the influence of SAD/ASD symptoms, we anticipated unstandardized effect sizes of 0.025, 0.03 and 0.03 for the main effects of distraction condition (surprised and warned) and symptom level, and 0.035 and 0.04 for the distraction by symptom level interactions. The random intercept variance and residual variance were assumed to be 0.05 and 0.09, respectively.

2. Previous literature (e.g., Dotov et al., 2016) indicates that the shape of a walking path can perturb gait structure. Given the shape of the alternative path was an irregular oblong (cf. a straight path for the other 95 participants), the data from these participants were excluded from the analysis to ensure consistency across the sample.

3. The LSAS has demonstrated good test–retest reliability (*r* = .83, *p* < .01), strong internal consistency (Cronbach’s α = .95), and convergent validity with the clinician-administered version (*r* = .83, *p* < .01; Baker et al., 2002). Guidelines suggest scores above 30 may indicate SAD (Mennin et al., 2002). The AQ-S has demonstrated good internal consistency reliability (Cronbach’s α = .77–.86), strong convergent validity with the 50-item AQ (*r* = .93–.95, *p* < .001) (Hoekstra et al., 2011) and test–retest reliability of *r* = .83 (Polderman et al., 2013). AQ scores above 65 are considered indicative of a clinically relevant level of autistic traits (Hoekstra et al., 2011).

4. To ensure consistency across walking trials, participants completed the no distraction trial whilst wearing the headphones, however, the headphones were switched off, and no audio could be heard through them.

5. Participants were initially given a free recall test in which they were asked to say aloud as many of the words they heard as possible (English or Macedonian) in 60s, followed by a comprehension test whereby the experimenter verbalised five Macedonian words (i.e., chaj, lubénitsa, studé, kapa, sína) and the participant was asked to recall the corresponding English translation (i.e., tea, watermelon, cold, hat, blue). Recall was compared between the surprised and warned conditions using Mann–Whitney U tests for the free recall and verbal comprehension tests separately. As expected, recall was poorer in the surprised (vs. warned) condition for both the free recall (*W* = 120.50, *p* < .001, *Mdn*_surprised_ = 6.00, *Mdn*_warned_ = 15.00) and comprehension tests (*W* = 264.00, *p* < .001, *Mdn*_surprised_ = 1.00, *Mdn*_warned_ = 3.00), indicating that the manipulation impacted recall as intended.

6. The resulting timeseries ranged in length from 4 min 11 s to 7 min 8 s (25,096–42,817 samples). No downsampling took place prior to analysis.

7. In response to a reviewer request, we also considered effects on MeanLine. These were consistent with the analysis of pMaxLine. Full analyses can be found in the Supplemental Materials (see Tables S5–S10).

8. The model was structured in this manner as the recall condition instructions (i.e., surprised vs. warned) were not provided until after the no distraction/baseline condition was completed.

9. Initially, we attempted to fit more complex random-effects structures that included random slopes for condition; however, these models failed to converge. We therefore report the maximally converging random-intercept only models.

10. %DET and pMaxL and were normalised via Fisher’s z transformation and log transformation, respectively.

11. Correlations between AQ scores and performance on the recall tests (i.e., manipulation checks) indicated clear trends towards negative associations for participants told to ignore the distraction (free recall test: ρ = −.262, *p* = .090, verbal comprehension test: ρ = −.220, *p* = .157), but no effects for those in the warned condition (free recall test: ρ = −.008, *p* = .954, verbal comprehension test: ρ = .091, *p* = .531). There were no equivalent effects when considering LSAS scores and performance on the recall test (all *p*’s > .5).
