# Supplementary material for: Subclinical Variation in Mental Health Shapes the Dynamics of Everyday Behaviour
Source: Q J Exp Psychol (Hove). 2026 Feb 5;79(9):2365–80. doi: 10.1177/17470218261424186 (PMC13498737; doi:10.1177/17470218261424186)
Supplement: Supplementary file 1 [file sj-docx-1-qjp-10.1177_17470218261424186.docx]

Supplementary materials for:

**Subclinical variation in mental health shapes the dynamics of everyday behaviour**

A. J. Brown^1^, M. C. Macpherson^1,2^ & L. K. Miles^1^

^1^ School of Psychological Science, University of Western Australia

^2^ School of Psychological Sciences, Macquarie University

Corresponding author:

Amber Brown

School of Psychological Science

University of Western Australia

35 Stirling Highway, Crawley

Perth WA 6009

Australia

Email: amber.brown@research.uwa.edu.au

Phone: +61439776152

| **Table S1.**  Distractor task word list | | |
| --- | --- | --- |
| **English** | **Macedonian** | **Phonetic spelling** |
| Tomato | Dómati | *DOH-mahti* |
| Hat | Kapa | *kah-PAH* |
| Blue | Sína | *SEE-nah* |
| Dog | Kúche | *KOO-cheh* |
| Pencil | Móliv | *MOH-leev* |
| Green | Zeléna | *zeh-LEH-nah* |
| Flower | Cveke | *SVE-keh* |
| Book | Bookor | *BOO-kor* |
| Light | Lightor | *li-TOR* |
| Salt | Sol | *SOHL* |
| Cake | Kekzar | *Kek-ZAR* |
| Onion | Krómid | *KROH-meed* |
| Soap | Sapún | *sah-POON* |
| Bag | Tórba | *TOR-bah* |
| Cold | Studé | *stoo-DEH* |
| Towel | Riza | *ri-ZAH* |
| Beard | Bráda | *BRAH-dah* |
| Red | Tsrvéna | *tsr-VEH-nah* |
| Mirror | Glédalo | *GLEH-dah-loh* |
| Dress | Fústán | *FOOS-tahn* |
| Tea | Chaj | *CHAI* |
| Money | Pári | *PAH-ree* |
| Chair | Stol | *STOHL* |
| Watermelon | Lubénitsa | *loo-BEH-neet-sah* |
| Olive | Máslinka | *MAHS-leen-kah* |

| **Table S3.**  Summary of the estimated marginal means from models examining the influence of condition on each metric of behavioural dynamics (i.e., %REC, %DET, pMaxL, α) | | | | | |
| --- | --- | --- | --- | --- | --- |
| **DV** | **Condition** | **Mean** | **SE** | ***df*** | **95% CI** |
| %REC | No distraction | –0.082 | 0.103 | 137 | [–0.29, 0.12] |
|  | Warned | 0.173 | 0.129 | 178 | [–0.08, 0.43] |
|  | Surprised | –0.025 | 0.137 | 182 | [–0.30, 0.25] |
|  |  |  |  |  |  |
| %DET | No distraction | –0.020 | 0.104 | 110 | [–0.23, 0.19] |
|  | Warned | 0.056 | 0.118 | 152 | [–0.18, 0.29] |
|  | Surprised | –0.022 | 0.123 | 162 | [–0.26, 0.22] |
|  |  |  |  |  |  |
| pMaxL | No distraction | 0.092 | 0.104 | 166 | [–0.11, 0.30] |
|  | Warned | –0.077 | 0.138 | 183 | [–0.35, 0.20] |
|  | Surprised | –0.108 | 0.148 | 183 | [–0.40, 0.18] |
|  |  |  |  |  |  |
| Alpha (α) | No distraction | 0.130 | 0.103 | 143 | [–0.07, 0.33] |
|  | Warned | –0.186 | 0.131 | 180 | [–0.44, 0.07] |
|  | Surprised | –0.065 | 0.140 | 183 | [–0.34, 0.21] |

| **Table S2.**  Summary of the fixed effects from models examining the influence of distraction on each measure of behavioural dynamics (i.e., %REC, %DET, pMaxL, α) | | | | | | |
| --- | --- | --- | --- | --- | --- | --- |
| **DV** | **Predictors** | **β** | **SE** | ***df*** | ***t*** | ***p*** |
| %REC | Distraction | 0.163 | 0.094 | 92 | 1.741 | .085 |
|  |  |  |  |  |  |  |
| %DET | Distraction | 0.040 | 0.063 | 92 | 0.638 | .525 |
|  |  |  |  |  |  |  |
| pMaxL | Distraction | −0.183 | 0.120 | 92 | −1.526 | .130 |
|  |  |  |  |  |  |  |
| Alpha (α) | Distraction | −0.260 | 0.100 | 92 | −2.603 | .011 |

| **Table S4.**  Summary of the pairwise comparisons from models examining the influence of condition on each metric of behavioural dynamics (i.e., %REC, %DET, pMaxL, α) | | | | | | |
| --- | --- | --- | --- | --- | --- | --- |
| **DV** | **Contrast** | **Estimate** | **SE** | ***df*** | ***t*** | ***p*** |
| %REC | No distraction – Warned | –0.254 | 0.122 | 108 | –2.088 | .097 |
|  | No distraction – Surprised | –0.057 | 0.130 | 111 | –0.438 | .900 |
|  | Warned – Surprised | 0.197 | 0.168 | 136 | 1.176 | .470 |
|  |  |  |  |  |  |  |
| %DET | No distraction – Warned | –0.076 | 0.084 | 98.7 | –0.906 | .638 |
|  | No distraction – Surprised | 0.002 | 0.090 | 100.1 | 0.021 | 1.000 |
|  | Warned – Surprised | 0.078 | 0.120 | 109.3 | 0.648 | .794 |
|  |  |  |  |  |  |  |
| pMaxL | No distraction – Warned | 0.169 | 0.151 | 116 | 1.118 | .505 |
|  | No distraction – Surprised | 0.200 | 0.160 | 122 | 1.245 | .429 |
|  | Warned – Surprised | 0.031 | 0.197 | 165 | 0.157 | .987 |
|  |  |  |  |  |  |  |
| Alpha (α) | No distraction – Warned | 0.316 | 0.129 | 110 | 2.454 | .041 |
|  | No distraction – Surprised | 0.194 | 0.137 | 114 | 1.415 | .337 |
|  | Warned – Surprised | –0.121 | 0.175 | 142 | –0.692 | .768 |

| **Table S5.**  Fixed effects of distraction on MeanLine | | | | | | |
| --- | --- | --- | --- | --- | --- | --- |
| **DV** | **Predictors** | **β** | **SE** | ***df*** | ***t*** | ***p*** |
| MeanLine | Distraction | 0.120 | 0.071 | 92 | 1.691 | .094 |

| **Table S6.**  Summary of the estimated marginal means from a models examining the influence of  condition on MeanLine. | | | | | |
| --- | --- | --- | --- | --- | --- |
| **DV** | **Condition** | **Mean** | **SE** | ***df*** | **95% CI** |
| MeanLine | No distraction | –0.060 | 0.104 | 116 | [–0.266, 0.146] |
|  | Warned | 0.095 | 0.121 | 162 | [–0.144, 0.334] |
|  | Surprised | 0.019 | 0.127 | 171 | [–0.230, 0.269] |

| **Table S7.**  Summary of the pairwise comparisons from models examining the influence of condition on  MeanLine. | | | | | | |
| --- | --- | --- | --- | --- | --- | --- |
| **DV** | **Contrast** | **Estimate** | **SE** | ***df*** | ***t*** | ***p*** |
| MeanLine | No distraction – Warned | –0.155 | 0.094 | 101 | –1.643 | .233 |
|  | No distraction – Surprised | –0.079 | 0.101 | 103 | –0.784 | .714 |
|  | Warned – Surprised | –0.076 | 0.134 | 115 | –0.565 | .839 |

| **Table S8.**  Summary of the estimated marginal trends from models examining the influence of condition and LSAS/AQ-S  on MeanLine. | | | | | | | |
| --- | --- | --- | --- | --- | --- | --- | --- |
| **DV** | **Condition** | **B** | **SE** | ***df*** | **95% CI** | ***t*** | ***p*** |
| *Liebowitz Social Anxiety Scale (LSAS)* | | | | | | | |
| MeanLine | No distraction | –0.078 | 0.105 | 113 | [ –0.285, 0.129] | –0.747 | .457 |
|  | Warned | –0.129 | 0.117 | 148 | [ –0.359, 0.101] | –1.107 | .270 |
|  | Surprised | –0.190 | 0.138 | 178 | [ –0.462, 0.082] | –1.379 | .170 |
|  |  |  |  |  |  |  |  |
| *Autism Spectrum Quotient-short form (AQ-S)* | | | | | | | |
| MeanLine | No distraction | –0.301 | 0.101 | 117 | [ –0.500, –0.101] | –3.274 | .004 |
|  | Warned | –0.237 | 0.114 | 154 | [ –0.462, –0.012] | –2.078 | .039 |
|  | Surprised | –0.280 | 0.132 | 177 | [ –0.540, –0.020] | –2.124 | .035 |

| **Table S9.**  Summary of the pairwise comparisons from models examining the influence of condition and LSAS/AQ-S on  MeanLine. | | | | | | | |
| --- | --- | --- | --- | --- | --- | --- | --- |
| **DV** | **Contrast** | **B** | **SE** | ***df*** | **95% CI** | ***t*** | ***p*** |
| *Liebowitz Social Anxiety Scale (LSAS)* | | | | | | | |
| MeanLine | No distraction – Warned | 0.051 | 0.088 | 95.5 | [–0.159, 0.261] | 0.579 | .832 |
|  | No distraction – Surprised | 0.112 | 0.115 | 101.8 | [–0.161, 0.384] | 0.976 | .593 |
|  | Warned – Surprised | –0.061 | 0.140 | 111.3 | [–0.394, 0.273] | –0.434 | .902 |
|  |  |  |  |  |  |  |  |
| *Autism Spectrum Quotient-short form (AQ-S)* | | | | | | | |
| MeanLine | No distraction – Warned | –0.064 | 0.090 | 97.8 | [-0.277, 0.149] | –0.712 | .757 |
|  | No distraction – Surprised | –0.021 | 0.111 | 103.5 | [–0.285, 0.243] | –0.189 | .981 |
|  | Warned – Surprised | –0.043 | 0.138 | 114.9 | [–0.371, 0.285] | –0.310 | .949 |

| **Table S10.**  Main effects LSAS/AQ-S on MeanLine. | | | | | | |
| --- | --- | --- | --- | --- | --- | --- |
|  | **B** | **SE** | ***df*** | **95% CI** | ***t*** | ***p*** |
| Liebowitz Social Anxiety Scale (LSAS) | –0.132 | 0.099 | 95.4 | [–0.331, 0.066] | –1.325 | .188 |
| Autism Spectrum Quotient-short form (AQ-S) | –0.273 | 0.096 | 95.8 | [–0.463, –0.083] | –2.854 | .005 |
